# Integrating Care for Children, Young People and Their Families

**DOI:** 10.5334/ijic.4189

**Published:** 2018-06-13

**Authors:** John Eastwood

**Affiliations:** 1 The Royal Prince Alfred Hospital, Sydney Local Health District, Camperdown, NSW 2050, AU

**Keywords:** Integrated care, children, young people, families, sustainable development

That “children are our future” is becoming increasingly evident as science comes to better understand the intergenerational, epi-genetic, and developmental origins of health and disease. The complex nature of child development, family, school and community environments, and the interplay of social, psychological and biological mechanisms, makes the provision of nurturing and protective services across the age ranges difficult. Approaches required during pregnancy, for example, will be very different to those provided for young people in transition to adulthood.

All societies have thus developed institutions and practices to nurture and protect their young through the various stages of development and transition to adulthood. The organisation and coordination of those activities is usually robust and complex. For some children and their families they will require a number of simultaneous complex inputs. It could be argued that the potential complexity of inputs, as a result of the developmental nature of childhood, is not realised at any other stage of the life-course. This is because each stage of child and youth development has its own unique complexity. Those stages might be defined as: pregnancy and childbirth; infancy (0–12 months), early childhood (0–4 years); middle childhood (5–9 year); adolescence (10–19 years), or early adulthood (20–29 years). The age related definitions are usually defined by demographers, legislators, and service planners, and often vary across service sectors, regions and jurisdictions. There are thus often challenges posed to the provision of seamless services across important developmental transition points, such as from childhood to adolescence, or adolescence to early adulthood with increased fragmentation of care occurring.

To function well the systems usually rely on informed participation of parents, caregivers, children and young people themselves. Children and young people, from about age 3, can be informed and have an active participation in the provision of services. Their involvement can be as active participants in clinical consultations, or through their involvement in planning and direct action to promote health, education and development in their own schools and communities.

The situation may be very different for families, children and young people living with current, or a previous history of adversity. The impact of that psychological trauma on physical and mental health has only recently been understood. The service system, its workforce, and institutions are often yet to adapt to this new knowledge and understanding. Consequently the behaviours of traumatised families, children and young people are not understood and services often fail those in most need of help. It is here that we are beginning to see the positive impact of “trauma informed” integrated health and social care approaches. Such systems require coordinated inputs from maternity services, childcare, schools, primary care, welfare and housing, income support, mental health, police, and hospitals. Population-based system-wide integrated care approaches utilise stratification of risk to ensure proportionate allocation of resources.

The integration of system-wide policies and services has long provided the foundation for promoting and protecting the health, development and wellbeing of children, young people and their families. The United Nations Convention on the Rights of Children and the Sustainable Development Goals (SDGs) provide the global platform to addressing the needs of children young people and families. The challenges are global. Integration of services for families, children and young people is important in low-income countries, countries affected by war, among refugee and migrant populations, and within rich countries as highlighted by the recent Innocenti Report “Building the Future: Children and the Sustainable Development Goals in Rich Countries” [1].

Integration of “whole of society” services for children, young people and their families has long had a role to play in low-income high-mortality countries. For much of the last 100 years health systems, governments and international agencies have worked to reduce maternal, infant and under-five mortality rates. Much of the improvement in mortality rates has been attributed to sanitation, clean water, fertility control, and improved access to primary health care that has included safe birthing practices and immunisation. In the face of slow gains in the development of community services in low-income countries, the world community signed the Alma Ata Declaration on Primary Health Care in 1978, and called for, among other things, local integration of services, and stronger community and interagency engagement.

Despite this primary health care focus on integration subsequent international “child survival” initiatives during the 1980s were characterised by a vertical and siloed approaches. Calls for stronger integration were met in the mid-1990s by the development by WHO and UNICEF of the Integrated Management of Childhood Illness (IMCI) strategy for implementation in countries with high child mortality rates. The IMCI strategy includes three components: (1) integrated management of ill children in facilities and health centres; (2) health system strengthening, particularly drugs and logistics support; and (3) community IMCI with a focus on promotion of key family and community practices. The IMCI strategy requires a strong partnership between health workers and families, with support from local communities. Community IMCI aims to reach families and communities in the place where they live. Thereby promoting and enabling the participation of parents, caregivers and communities in their own development.

In the current global health context, the United Nations Convention on the Rights of Children (UNCRC) and the Sustainable Development Goals (SDGs), together provide a global platform to addressing the needs of children young people and families through an integrated system response. Building on the SDG goal of achieving universal health coverage, WHO has developed a global strategy and *Framework for people-centered and integrated health services* [2], recommending that countries consciously consider the perspectives of individuals, families, and communities, and respond to their preferences and needs. The framework builds on the foundations of the earlier Alma Ata Declaration of Primary Health Care (and arguably the Ottawa Charter of Health Promotion), and proposes five interdependent strategies for health services to become more integrated and people-centred. They are: (1) empowering and engaging people and communities; (2) strengthening governance and accountability; (3) reorienting the model of care; (4) coordinating services within and across sectors; and (5) creating an enabling environment.

At heart of achieving the Sustainable Development Goals is also the Global Strategy for Women’s, Children’s and Adolescents’ Health (2016–2030) [3]. That strategy “envisions a world in which every woman, child and adolescent realizes their rights to physical and mental health and well-being, has social and economic opportunities, and is able to participate fully in shaping prosperous and sustainable societies”. Building on the “Global Strategy for Women’s, Children’s and Adolescents’ Health (2016–2030)”, a “Nurturing care for early childhood development” framework was recently launched at the 71st World Assembly [4]. That Framework contains five guiding principles: (1) the child’s right to survive and thrive (a Child Rights principal); (2) leave no child behind (an equity principal); (3) family-centred care; (4) whole-of-government action; and (5) a whole-of-society approach.

Based on an analysis of effective programmes the WHO Framework proposes five action areas “to empower families to provide nurturing care … [and] create enabling environments, strong monitoring systems and accountability mechanisms.” The five proposed action areas are to: (1) lead and invest; (2) focus on families and their communities; (3) strengthen services; (4) monitor progress; and (5) use data and innovate.

The two WHO frameworks for people-centered and integrated health services and Nurturing Care provide a useful basis to strengthen current international approaches to Integrating Care for Children Young People and their Families. At the recent International Conference on Integrated Care in Utrecht we called for the establishment of an IFIC special interest group (SIG) for “Integrating Care for Children Young People and their Families”. The SIG aims to bring together health, education and social care practitioners and researchers who are interested in aspects of integrated health and social care as it applies to children, young people and their families. As a group we will discuss service, policy and system approaches and collaborate on Integrated Care research and development projects, including grant proposals.

Our first steps will include: building a broad global network; establishing a community website; linking with other global networks, including Child Health Information for All (CHIFA); defining the definitions and scope of Integrated Health and Social Care as it applies to Children, Young People and their Families; preparing and publishing a joined position paper on ‘Integrated Care for Children Young People and their Families; collaborating on shared research projects; developing and promoting appropriate outcome measures and evaluation frameworks; sharing successes and lessons-learnt of Integrated Care as it applies to Children, Young People and their Families; and meet during the International Conferences on Integrated Care. There are challenges in evaluating outcomes and the complex interventions required for integration of care, and thus the required methodological approaches will be a key issue for early discussion within the SIG.

Through this global network we believe that we can share knowledge and experience that will contribute to advancing the vision of the *Health for All*. If you are interested in the IFIC Special Interest Group – Integrating Care for Children, Young People, and Their Families, please contact me at john.eastwood@health.nsw.gov.au.

## Author Information

Director Healthy Homes and Neighbourhoods Integrated Care Initiative, Director Community Paediatrics, Child & Family Clinical Services, Community Health, Executive Clinical Advisor – Clinical Services Integration and Population Health Sydney Local Health District, Camperdown, NSW, Australia.
